# BP-M345, a New Diarylpentanoid with Promising Antimitotic Activity

**DOI:** 10.3390/molecules26237139

**Published:** 2021-11-25

**Authors:** Pedro Novais, Patrícia M. A. Silva, Joana Moreira, Andreia Palmeira, Isabel Amorim, Madalena Pinto, Honorina Cidade, Hassan Bousbaa

**Affiliations:** 1 UNIPRO—Oral Pathology and Rehabilitation Research Unit, University Institute of Health Sciences (IUCS), CESPU, Rua Central de Gandra, 1317, 4585-116 Gandra, Portugal; pedro.ha.novais@gmail.com (P.N.); patricia.silva@cespu.pt (P.M.A.S.); 2 Faculty of Sciences, University of Porto, Rua do Campo Alegre, s/n, 4169-007 Porto, Portugal; 3 ICBAS—Instituto de Ciências Biomédicas Abel Salazar, University of Porto, Rua de Jorge Viterbo Ferreira 228, 4050-313 Porto, Portugal; 4 TOXRUN—Toxicology Research Unit, University Institute of Health Sciences, CESPU, CRL, 4585-116 Gandra, Portugal; 5 Laboratory of Organic and Pharmaceutical Chemistry, Department of Chemical Sciences, Faculty of Pharmacy, University of Porto, Rua de Jorge Viterbo Ferreira 228, 4050-313 Porto, Portugal; up201302558@edu.ff.up.pt (J.M.); apalmeira@ff.up.pt (A.P.); madalena@ff.up.pt (M.P.); 6 Interdisciplinary Centre of Marine and Environmental Research (CIIMAR), University of Porto, Edifício do Terminal de Cruzeiros do Porto de Leixões, Avenida General Norton de Matos, s/n, 4450-208 Matosinhos, Portugal; 7 GreenUPorto (Sustainable Agrifood Production) Research Center, Faculty of Sciences, University of Porto, Rua do Campo Alegre, s/n, 4169-007 Porto, Portugal; mpamorim@fc.up.pt

**Keywords:** diarylpentanoids, BP-M345, antitumor, mitosis, apoptosis

## Abstract

Previously, we reported the in vitro growth inhibitory effect of diarylpentanoid BP-M345 on human cancer cells. Nevertheless, at that time, the cellular mechanism through which BP-M345 exerts its growth inhibitory effect remained to be explored. In the present work, we report its mechanism of action on cancer cells. The compound exhibits a potent tumor growth inhibitory activity with high selectivity index. Mechanistically, it induces perturbation of the spindles through microtubule instability. As a consequence, treated cells exhibit irreversible defects in chromosome congression during mitosis, which induce a prolonged spindle assembly checkpoint-dependent mitotic arrest, followed by massive apoptosis, as revealed by live cell imaging. Collectively, the results indicate that the diarylpentanoid BP-M345 exerts its antiproliferative activity by inhibiting mitosis through microtubule perturbation and causing cancer cell death, thereby highlighting its potential as antitumor agent.

## 1. Introduction

Microtubule-targeting agents (MTAs) remain one of the best characterized therapeutic drugs for the treatment of a wide range of tumor types, including ovarian, lung, breast, head, and neck [1]. MTAs are typically divided into two groups based on their action mechanism: microtubule destabilizers and stabilizers [2,3]. Microtubule destabilizers consist of vinca alkaloids, such as vinblastine and vincristine, that bind to β-tubulin subunits, inducing microtubule depolymerization. Microtubule stabilizers include epothilones and taxanes, such as paclitaxel and docetaxel, and bind to β-tubulin subunits, resulting in the stabilization of microtubules and increased polymerization [3,4].

By perturbing the mitotic spindle, MTAs activate the spindle assembly checkpoint (SAC), which delays cancer cells in mitosis. The SAC is a highly sensitive mechanism that controls chromosome attachment at kinetochores to spindle microtubules [5]. In the presence of unattached or mis-attached kinetochores, the SAC promotes the generation of the mitotic checkpoint complex (MCC), formed by the association of BUB3, MAD2, and BUBR1 proteins with CDC20, a co-factor of the anaphase promoting complex/cyclosome (APC/C), resulting in mitotic arrest. Once all chromosomes have established a bipolar attachment with spindle microtubules and become aligned at metaphase plate, the MCC complex disintegrates and releases CDC20 protein, which is able to bind and activate the APC/C, which leads to mitosis exit and cell cycle progression [6,7,8].

Despite the effectiveness of MTAs to prevent cancer cells from dividing, some cancers become insensitive or resistant to them. In addition, several side effects, including myelo- and neurotoxicity, are reported [9,10,11]. Additionally, under MTA treatment, mitosis-arrested cancer cells can experience different fates. Ideally, cancer cells die during the mitotic arrest. However, cancer cells can exit from mitosis without cell division in a process called mitotic slippage, which may lead to more genomic instability, thereby increasing tumor aggressiveness [12,13]. Slippage is considered an important mechanism of resistance to MTAs. Therefore, new MTAs that can overcome the disadvantages associated with the MTAs currently in use in the clinic are needed, and their identification is a very active area of research [14,15,16].

**Figure 1:**
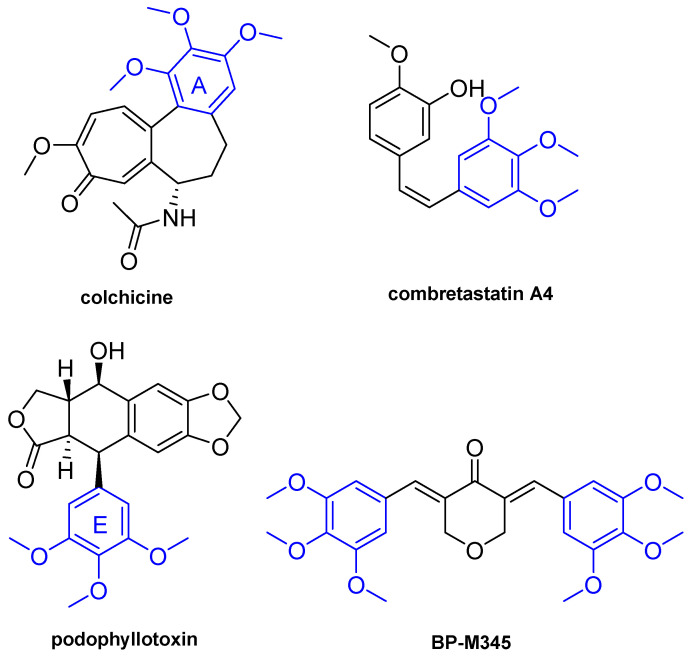
Chemical structures of colchicine, combretastatin A4, podophyllotoxin and diarylpentanoid BP-M345.

The presence of a 3,4,5-trimethoxyphenyl group in some MTAs, such as colchicine, combretastatin A4, and podophyllotoxin (Figure 1), has been much highlighted as important for the interaction with tubulin [17]. In fact, several compounds with a 3,4,5-timethoxyphenyl fragment, such as trimethoxybenzyl alcohol and trimethoxybenzaldehyde, have been evaluated for binding with tubulin, and it has been demonstrated that these compounds inhibit the binding of colchicine to tubulin through interaction with the colchicine binding site [18].

Diarylpentanoids comprise a class of natural products and their synthetic analogues, structure related with chalcones, possessing two aromatic rings connected by a five-carbon bridge. These compounds have gained increasing interest over the last few decades due to a wide variety of biological activities, including antioxidant [19], anti-inflammatory [20], antitumor [21], antidiabetic [22], antibacterial [23], antiparasitic [24], and antihyperuricemic [25]. In addition, diarylpentanoids have also been described for their ability to interfere with several proteins, namely enzymes [26,27,28,29,30,31,32] (oxidoreductases, glycosidases, phosphatases, cholinesterases, and carbonic anhydrase II), and channel proteins (P-gp, BCRP, MRP1, MRP5, TRPA1, TRPM8, and TRPV1). The antitumor potential of diarylpentanoids has been widely studied, having identified several compounds as potent in vitro growth inhibitors of human tumor cell lines, with GI_50_ values of nanomolar range [21]. However, the underlying molecular mechanism by which these compounds suppress cancer cell growth is still unclear.

Previously, the diarylpentanoid BP-M345 (Figure 1) has been identified as a potent in vitro growth inhibitor of human colon cancer HCT116 cells expressing wt p53, with a GI_50_ value of 0.17 µM, showing low toxicity in non-tumor HFF-1 cells [33]. Taking this into account, and aiming to continue the search for new p53-MDM2 inhibitors by our research group [34,35,36], the possible effect of BP-M345 on p53 pathway was investigated. Particularly, the potential activation of wild-type p53 by inhibiting its interaction with MDM2 was evaluated using a yeast approach [37]. The results obtained showed that BP-M345 has no effect on the inhibition of the p53-MDM2 interaction [33]. Thus, considering that BP-M345 possesses 3,4,5-trimethoxyphenyl groups that have been much highlighted as playing crucial role in the interaction with tubulin in MTAs, such as podophyllotoxin, combretastatin A4, and colchicine [38], as well as some chalcones previously reported as potent antimitotic agents by our group [38,39,40], it was hypothesized that this diarylpentanoid could also act as antimitotic agent.

The purpose of this study is to investigate the potential antimitotic activity of BP-M345 and the underlying mechanism by which it promotes cancer cell death.

## 2. Results

### 2.1. Compound BP-M345 Exhibits a Potent Tumor Growth Inhibitory Activity with High Selectivity Index

In order to evaluate the potential of BP-M345 to inhibit tumor cell growth, a Sulforhodamine B (SRB) assay was performed to determine the concentration of a compound that induces 50% cell growth inhibition (GI_50_) in three human cancer cell lines from melanoma (A375-C5), breast adenocarcinoma (MCF-7), and non-small cell lung cancer (NCI-H460). BP-M345 demonstrated a potent growth inhibitory activity in all tested cancer cell lines, with a GI_50_ ranging from 0.24 μM to 0.45 μM (Table 1 and Figure S1).

Additionally, the GI_50_ of BP-M345 in the non-tumor human pulmonary alveolar epithelial cells (HPAEpiC) was 1.07 ± 0.16 μM, more than two-fold higher than in the tumor cells, suggesting that BP-M345 is less toxic to non-tumor cells, at least at concentrations that are toxic to tumor cells. Importantly, selectivity index calculation (Table 2) showed a notable degree of selectivity of the compound BP-M345 in all cell lines studied. These results were in agreement with those previously reported by our research group in colon cancer HCT116 cells and non-tumor HFF-1 cells [33]. Interestingly, the selectivity index of BP-M345 demonstrated a better degree of selectivity than doxorubicin.

These results reinforced the potential of BP-M345 as a promising and selective anticancer agent that led us to further investigate the mechanism underlying its cytotoxicity.

### 2.2. Compound BP-M345 Arrests Tumor Cells at Mitosis

**Figure 2:**
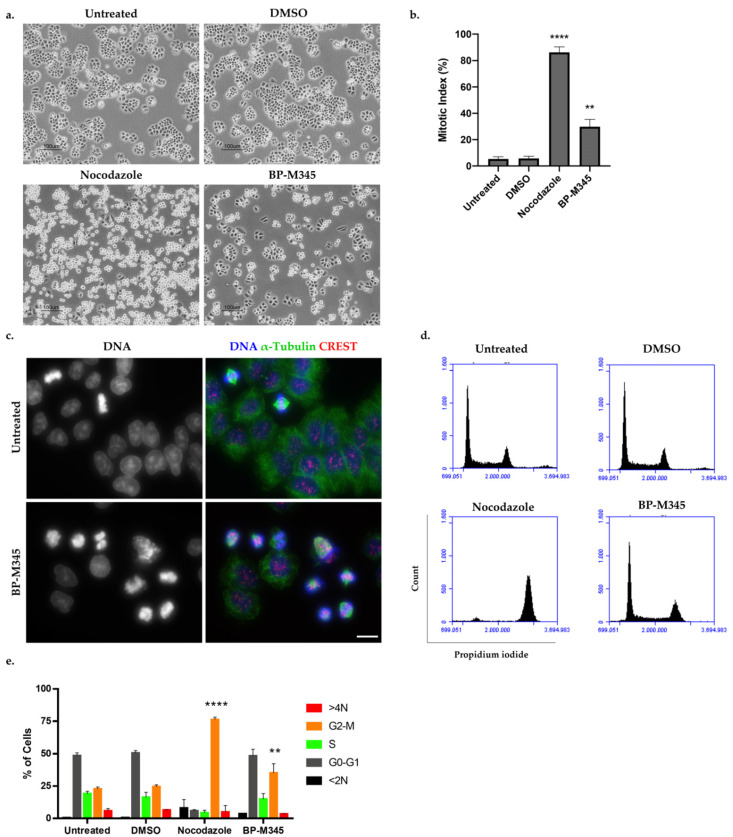
Treatment with BP-M345 induces mitotic arrest of tumor cells. (**a**) Representative phase contrast microscopy images of untreated cells and cells treated with 0.74 μM of BP-M345, for 16 h, showing accumulation of rounded and bright cells (mitotic cells). Cells treated with DMSO and nocodazole were used as controls. Bar, 100 µm. (**b**) Mitotic index of data shown in A with statistical relevance of ** *p* < 0.01 and **** *p* < 0.0001 by unpaired t-test from three independent experiments. (**c**) Immunofluorescence images of untreated and BP-M345-treated tumor cells, confirming the presence of mitotic cells by DNA condensation and mitotic spindle. Microtubules (green) were stained with anti-α-tubulin antibody, kinetochores (red) with anti-CREST antibody, and DNA (blue) with DAPI. Bar, 5 µm. (**d**) Representative flow cytometry histogram for cell cycle distribution using propidium iodide (PI) staining after exposure of cells to BP-M345 for 16 h. (**e**) Bar graphic showing the percentage of cell cycle stages (G0/G1, S G2/M) and <2N and 4N demonstrating an increase in cells in G2/M phase with statistical relevance of ** *p* < 0.01 and **** *p* < 0.0001 by two-way ANOVA with Tukey’s multiple comparisons test from three independent experiments.

To unveil the cytotoxic mechanism of BP-M345, we selected the NCI-H460 cell line due to its suitability for quantitative evaluation of morphological changes. We first analyzed cells treated with BP-M345 for 16 h by phase contrast microscopy (Figure 2a). Untreated cells and cells treated with Dimethyl sulfoxide (DMSO), the compound solvent, were used as controls. We found an accumulation of bright and round cells, reminiscent of mitotic cells, under BP-M345 treatment, that mirrored nocodazole-treated cells, an antimitotic agent used as a positive control (Figure 2a). Calculation of the mitotic index (MI) revealed a significant increase in MI in BP-M345-treated cells (29.8 ± 5.6%), compared to the untreated (5.3 ± 1.8%) or DMSO-treated (5.7 ± 1.7%) cells (Figure 2b). Additionally, we performed an immunofluorescence assay under the same treatment conditions, in which the accumulation of mitotic cells was confirmed, as demonstrated by the DNA condensation after DAPI staining, and by the presence of the mitotic spindle after microtubule staining with an anti-α-tubulin antibody (Figure 2c). We further investigated the cell cycle distribution by flow cytometry of the BP-M345-treated cells (Figure 2d). We noticed that the percentage of cells in the G2/M phase was significantly higher upon treatment with BP-M345 (35.2 ± 7.0%) compared to untreated (22.75 ± 1.5%) or DMSO-treated cells (24.56 ± 1.4%), thereby corroborating the results above obtained by contrast microscopy and immunofluorescence analyses. Taken together, these results suggest that the compound BP-M345 exerts its anti-growth activity by acting as an antimitotic agent.

### 2.3. Compound BP-M345 Affects Mitotic Spindle Morphology and Disturbs Chromosome Congression

**Figure 3:**
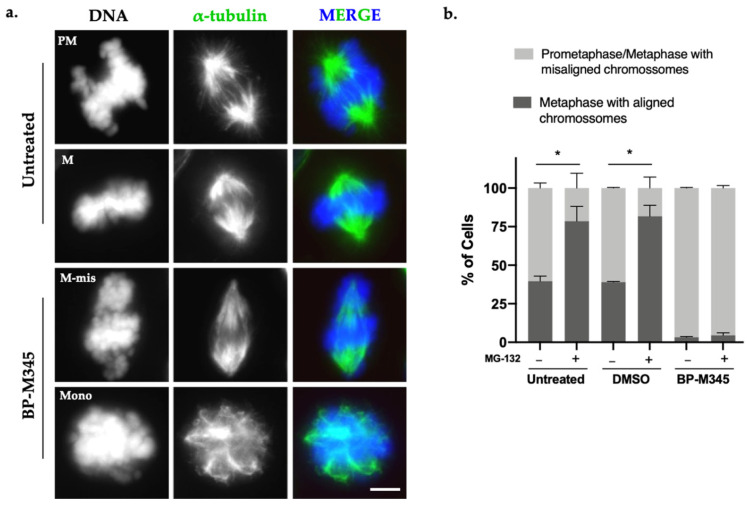
BP-M345 treatment disturbs mitotic spindle morphology. (**a**) Immunofluorescence images of untreated cells and cells treated with 0.74 μM of BP-M345, showing monopolar spindle configuration (Mono) and metaphase with misaligned chromosomes (M-mis) after the treatment, compared to bipolar spindle from untreated cells, in prometaphase (PM) and metaphase (M). Microtubules (green) were stained with anti-α-tubulin antibody and DNA (blue) with DAPI. Bar, 5 µm. (**b**) Graphical representation of metaphases with misaligned vs. aligned chromosomes, in absence (−) or presence (+) of proteasome inhibitor MG-132 at indicated conditions with statistical relevance of * *p* < 0.05 by two-way ANOVA with Tukey’s multiple comparisons test from three independent experiments.

During the immunofluorescence analysis of BP-M345-treated cells, we observed that the majority of cells exhibited several misaligned chromosomes and mitotic spindles with abnormal morphology (Figure 3a), including spindles with decreased microtubule density, and some monopolar spindles, contrasting with the robust and bipolar spindle of control cells (Figure 3a). This suggested that BP-M345 was disturbing the mitotic spindle assembly. To find out whether the chromosome misalignment was irreversible or could be corrected over time, we quantified the number of full metaphases and metaphases with misaligned chromosomes in the presence or absence of the proteosome inhibitor MG-132. The MG-132 blocks the cells at metaphase-to-anaphase transition by preventing the proteasome-mediated degradation of cyclin B and securin [41,42]. This way, by treating the cells with MG-132, cells have more time to align all chromosomes at the equatorial region before anaphase onset. We observed that, in untreated and DMSO-treated cells, the percentage of metaphases with all chromosomes aligned was significantly increased after addition of MG-132 (52.2% to 78.5% and 49.7% to 81.8%, respectively) (Figure 3b). Meanwhile, in BP-M345-treated cells, the large majority of cells continued to exhibit misaligned chromosomes even after addition of MG-132 (96.7% and 95.5% before and after addition of MG-132, respectively), suggesting that chromosome misalignment was a permanent defect that could not be corrected over time (Figure 3b). Taken together, these results indicate that the compound BP-M345 disturbs the mitotic spindle and irreversibly induces defects in chromosome congression.

### 2.4. BP-M345 Interferes with the Stability of Kinetochore-Microtubule Attachments and Promotes SAC Activation

**Figure 4:**
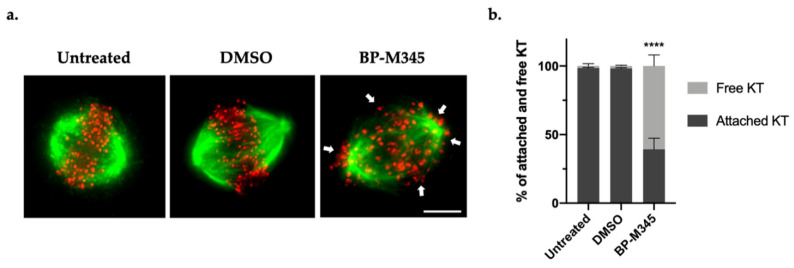
Treatment with BP-M345 interferes with the stability of kinetochore-microtubule attachments. (**a**) Representative immunofluorescence images after cold treatment assay, showing several unattached kinetochores (free red spots pointed by the white arrowheads) in cells treated with 0.74 μM of BP-M345 treatment, whereas most kinetochores were attached (Red spots with attached green fibers) in untreated cells. Microtubules (green) were stained with anti-α-tubulin antibody, kinetochores (red) with anti-CREST antibody, and DNA (blue) with DAPI. (**b**) Quantification of cold-stable microtubules (as percentage of attached kinetochores per cell) after treatment with BP-M345 with statistical relevance of **** *p* < 0.001 by two-way ANOVA with Tukey’s multiple comparisons test from three independent experiments.

As robust kinetochore-microtubule (KT-MT) attachments are required for chromosome congression, and because BP-M345 was inducing persistent misaligned chromosomes, we wondered if it was interfering with the stability of the KT-MT attachments. To evaluate this hypothesis, we performed a classic cold treatment assay. Unstable or weakened KT-MT attachments are disassembled when exposed to low temperature for a short time period, while stable and robust attachments (K-fibers) are resistant to this condition [43]. Thus, we subjected the cells to 4 °C for 5 min, under MG-132 treatment, and then we performed an immunofluorescence staining using a human anti-CREST (Raynaud’s phenomenon, esophageal dysmotility, sclerodactyly, and telangiectasias) antibody to localize the kinetochores, and an anti-α-tubulin antibody to visualize the spindle microtubules. In untreated and DMSO-treated cells, almost all KTs remained attached to MTs (98.6 ± 0.4% and 98.2 ± 0.6%, respectively) (Figure 4a,b), while BP-M345-treated cells showed few cold-stable KT-MT attachments (39.2 ± 8.1%) and several free kinetochores (60.8 ± 8.1%) (Figure 4a,b). This result suggests that the BP-M345 weakens KT-MT attachments, compromising chromosome congression, which results in the aforementioned persistent chromosome misalignment phenotype.

**Figure 5:**
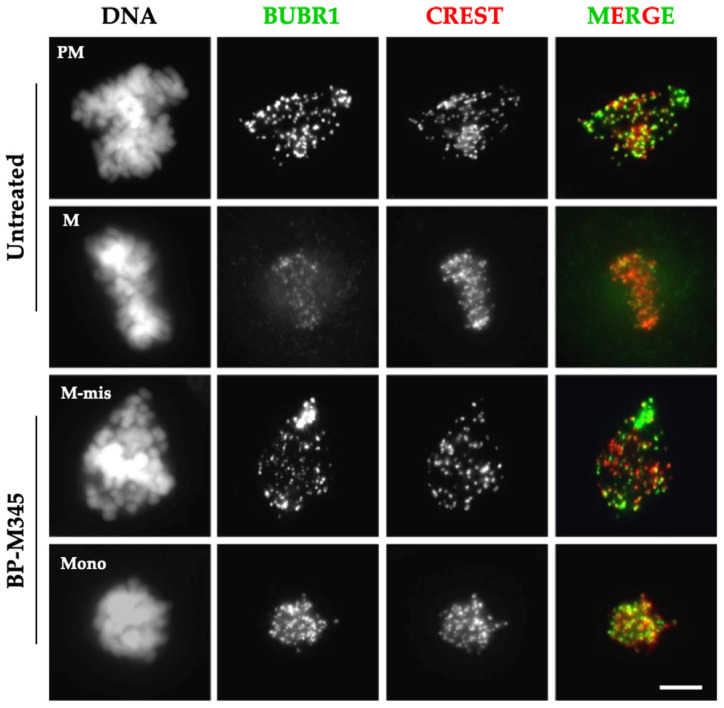
Treatment with BP-M345 leads to spindle assembly checkpoint activation. Representative immunofluorescence staining from three independent experiments using antibodies against BUBR1 (green) and CREST (red) in untreated cells and in cells treated with 0.74 μM of BP-M345. DAPI was stained with DAPI. In untreated cells, BUBR1 strongly localizes at kinetochores, in prometaphase (PM) and clearly decreases in metaphase (M) consistent with their normal localization pattern. In BP-M345-treated cells (bottom panel), BUBR1 is present in all mitotic cells, either with misaligned chromosomes (M-mis) or with monopolar spindles (Mono), indicating mitotic checkpoint activation. Bar, 5 µm.

We then investigated whether BP-M345 was promoting the activation of the spindle assembly checkpoint (SAC) to sustain the observed mitotic arrest. The SAC is responsible for blocking metaphase-to-anaphase transition, until all chromosomes are correctly attached to spindle microtubules through their kinetochores and aligned at the metaphase plate. Through mitotic checkpoint complex (MCC) assembly, the SAC ultimately inhibits the anaphase-promoting complex/cyclosome (APC/C) preventing mitosis progression. The kinase BUBR1 is a component of MCC, and it accumulates at kinetochores when SAC is on. When kinetochores become correctly attached to microtubules, and all chromosomes are aligned at the metaphase plate, the BUBR1 leaves the kinetochores during the process of MCC disassembly and SAC silencing [7]. Thus, the presence of BUBR1 at kinetochores is a good marker of SAC activation. We thus performed immunofluorescence staining using antibodies against BUBR1 and kinetochores (CREST) in untreated and BP-M345-treated cells. As expected, in untreated cells at metaphase, BUBR1 localization at kinetochores was drastically reduced, as judged by the weak staining, indicating SAC inactivation (Figure 5). In contrast, in BP-M345-treated cells, a strong and bright BUBR1 staining was detected in all kinetochores, demonstrating that the SAC was activated in treated cells (Figure 5). Taken together, these results suggest that BP-M345 compromises the stability of KT-MT attachments and chromosome congression, which leads to a chronic activation of the SAC, thereby inducing cell arrest at mitosis.

### 2.5. Fate of BP-M345-Treated Cells Arrested in Mitosis

**Figure 6:**
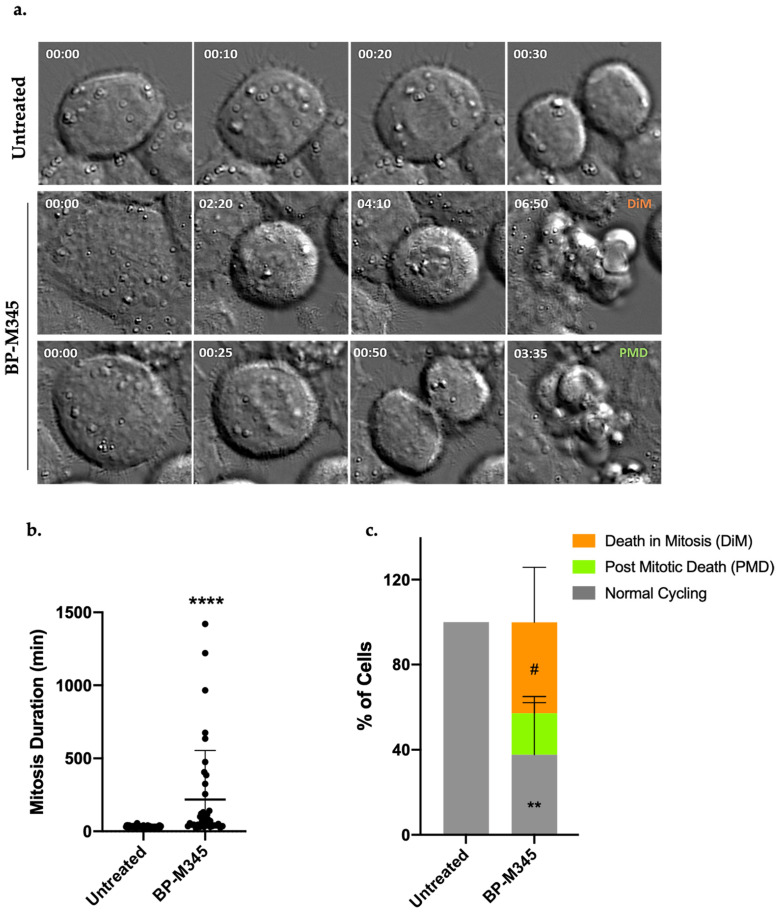
BP-M345 promotes tumor cell death, mostly in mitosis. (**a**) Representative time-lapse sequences of untreated cells and cells treated with 0.74 μM of BP-M345. Untreated cells undertake mitosis in (31.8 ± 5.6 min, **top**), while BP-M345-treated cells arrest in mitosis (218.7 ± 335.6 min, **bottom**) followed by death in mitosis or post-mitosis. (**b**) Mitosis duration as determined by time-lapse microscopy in untreated (N = 57) and BP-M345-treated cells (N = 39) from three independent experiments showing an increase in time spent in mitosis of tumor cells with statistical relevance of **** *p* < 0.0001 by unpaired t-test. Each spot represents one cell. (**c**) Quantification of cell fate after treatment with BP-M345. Representation of the percentage of cells undergoing post-mitotic death (PMD) and death in mitosis (DIM), and cells with normal cycling, from three independent experiments with statistical relevance of ** *p* < 0.01 (normal cycling) and # *p* < 0.05 (death in mitosis) by two-way ANOVA with Tukey’s multiple comparisons test.

In order to investigate the fate of the cells that were arrested in mitosis under BP-M345 treatment, we performed a live-cell imaging using time lapse differential interference contrast (DIC) microscopy (Figure 6a) during 48 h. We firstly found that BP-M345-treated cells remain in mitosis for 218.7 ± 335.6 min, almost 7-fold longer than untreated cells (31.8 ± 5.6 min) (Figure 6b). Fate analysis of mitotic cells showed that 42.7 ± 24.9% of BP-M345-treated cells died in mitosis (Video S2), and 19.5 ± 7.8% (Video S3) underwent post-mitotic death, while 37.7 ± 24.5% were able to continue dividing (Figure 6c, Video S1).

**Figure 7:**
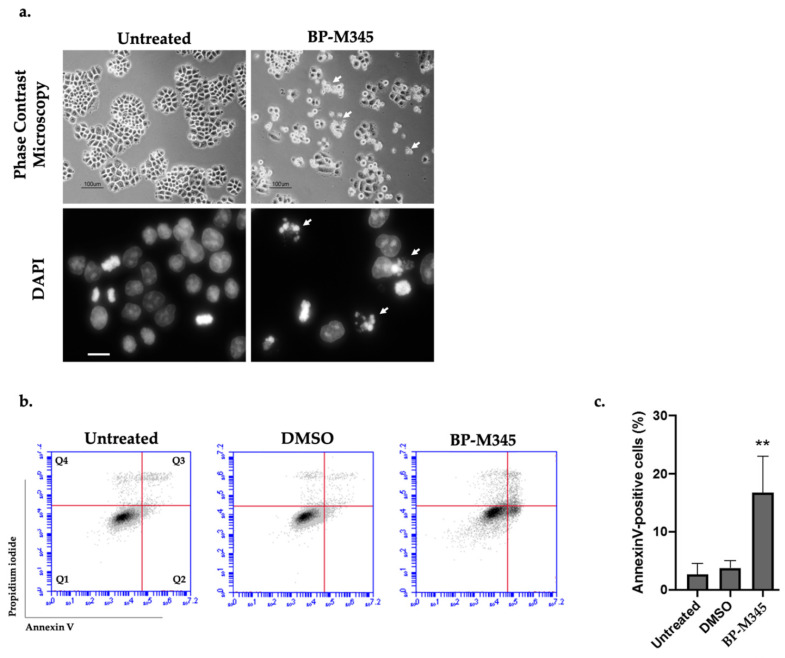
Tumor cells treated with BP-M345 undergo apoptotic cell death. (**a**). Representative phase contrast microscopy (**Top**) and DAPI staining (**bottom**) images of untreated cells and cells treated with 0.74 μM of BP-M345 after 24 h exposure, showing accumulation of floating (arrows) and condensing cells (arrows), respectively, suggesting cell death. (**b**). Representative flow cytometry histogram of Propidium iodide (PI) versus Annexin V (FITC-A) intensity in untreated and BP-M345-treated tumor cells, at 24 h. DMSO was used as control. The quadrants Q were defined as Q1 = live cells (Annexin V- negative/PI-negative), Q2 = early stage of apoptosis (Annexin V-positive/PI-negative), Q3 = late stage of apoptosis (Annexin V-positive/PI-positive) and Q4 = necrosis (Annexin V-negative/PI-positive). (**c**). Quantification of AnnexinV-positive cells of the data shown in B with statistical relevance of ** *p* < 0.01 by unpaired t-test from three independent experiments.

We also evaluated the NCI-H460 tumor cells after 24 h treatment with BP-M345 by phase contrast microscopy and after DAPI staining (Figure 7a). By phase contrast microscopy analysis, we noted the presence of floating cells indicative of dead cells after treatment with BP-M345, in agreement with time-lapse analysis. By DAPI staining, we observed that several cells displayed micronucleation and morphology reminiscent of apoptosis. Thus, to confirm this observation, cells treated for 24 h with BP-M345 were stained with Annexin V/PI staining and analyzed by flow cytometry (Figure 7b). In untreated cells, the percentage of apoptosis was residual (2.7 ± 1.8%), while in BP-M345-treated cells, a significant increase in apoptotic cells was observed (16.8 ± 6.2%) (Figure 7c). These results indicate that, after mitotic arrest imposed by BP-M345 treatment, the cells die by apoptosis.

### 2.6. BP-M345 Exerts a Long-Term Inhibitory Effect on Tumor Cell Proliferation

**Figure 8:**
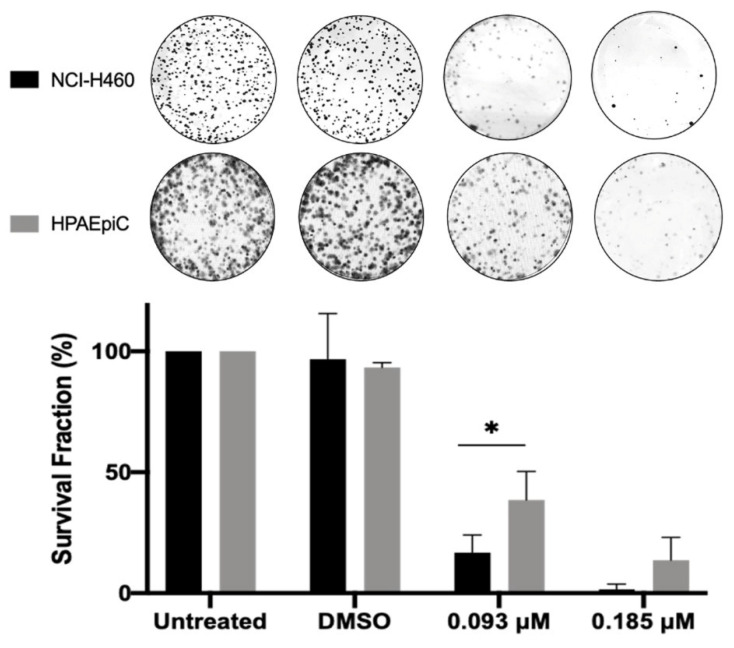
BP-M345 treatment compromises long-term tumor cell proliferation. NCI-H460 and HPAEpiC cells were treated with 0.185 and 0.093 μM of BP-M345 for 48 h. A 6-day colony-formation assay was performed. After washout, the surviving colonies were stained with crystal violet, and a representative figure is shown for each condition. Results are the mean of three independent experiments, expressed as % of survival fraction with statistical relevance of * *p* < 0.05 by two-way ANOVA with Tukey’s multiple comparisons test.

To evaluate the long-term effect of BP-M345 on tumor cell proliferation, we performed a colony formation assay. Cells were treated with 0.185 and 0.093 μM of BP-M345 (2- and 4-fold less than the GI_50_) for 48 h. Cells were then washed and incubated in a drug-free culture medium. After 6 days, the number of colonies formed was scored. As expected, untreated and DMSO-treated cells extensively proliferated, while cells treated with 0.185 μM of BP-M345 were unable to form colonies or only few colonies, approximately 16 ± 7.3%, under treatment with 0.093 μM of BP-M345 (Figure 8). Therefore, this result suggests that, although the time-lapse assay indicated that 43.6% of BP-M345-treated cells survived 48 h post-treatment, these cells would not be able to further proliferate beyond this treatment time, indicating that BP-M345 exerts a long-term inhibitory effect on cell proliferation. Importantly, at the highest concentration (0.185 μM) of BP-M345, which killed almost 100% of tumor cells, the lung non-tumor HPAEpiC cells showed less sensitivity to the treatment, with a survival fraction of 13% contrasting with 1.5% in cancer cells, indicating that the compound is more toxic to tumor cells when compared to non-tumor cells (Figure 8), possibly due to a higher proliferative rate of cancer cells compared to non-tumor cells.

### 2.7. Docking Studies

In order to further understand the mechanism of action of the diarylpentanoid BP-M345 as antimitotic agent, a docking study in the binding site of α,β-tubulin (PDB: 4O2B) was performed for this compound along with compounds already described in the literature as tubulin inhibitors [17]. AutoDock Vina was the software chosen to predict docking conformations and scores. The results for the positive controls colchicine, combretastatin A4, and podophyllotoxin and for the test compound BP-M345 are presented in Table 3.

**Figure 9:**
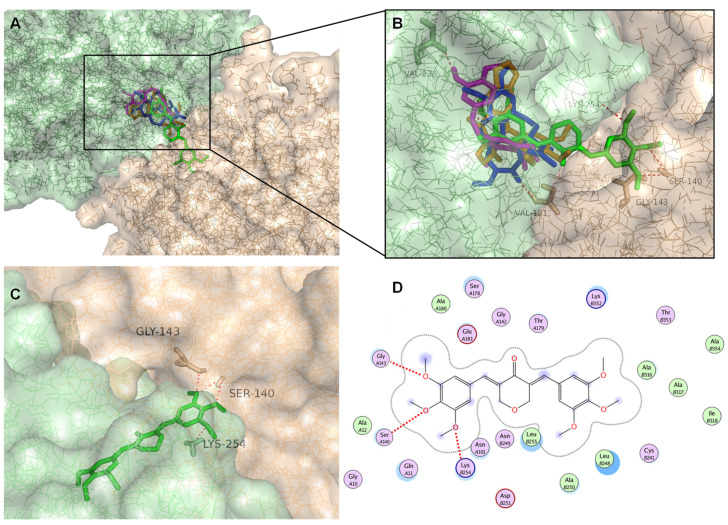
The molecular docking model of BP-M345, colchicine, combretastatin A4, and podophyllotoxin with tubulin. (**A**) All tested compounds docked in the interface between tubulin α (light orange) and β (light green) units. (**B**) Predicted binding poses of BP-M345 (green sticks), colchicine (blue sticks), combretastatin A4 (magenta sticks), and podophyllotoxin (orange sticks) in the binding site of tubulin (transparent solid surface). (**C**) Predicted binding poses of BP-M345 (green sticks) in the binding site of tubulin with depicted hydrogen interaction. Tubulin is represented as surface, where tubulin α and β are represented in light orange and light green, respectively. Hydrogen interactions are depicted with a dashed red line. Residues evolved in polar interactions in α- and β-tubulin are labelled and represented in orange or green, respectively. (**D**) 2D depiction of test compound BP-M345 in colchicine binding site. Hydrogen interactions are represented as red dashed lines. Receptor residues that are close to the ligand, but whose interactions with the ligand are weak or diffuse, such as collective hydrophobic or electrostatic interactions, are also represented (all the ones that have no indication for hydrogen-bonding). Solvent accessible surface area of the ligand is plotted directly onto the atoms in the form of a blue smudge. Solvent accessible surface area for the receptor residues is plotted as a blue halo. (For interpretation of the references to color in this figure legend, the reader is referred to the Web version of this article.).

Compound BP-M345 presented higher affinity to tubulin (−8.7 kcal mol^−1^) than the known inhibitors combretastatin A4 and podophyllotoxin (−7.9 and −8.4 kcal mol^−1^, respectively). Figure 9A,B show the binding conformations of crystallographic colchicine, combretastatin A4, podophyllotoxin, and BP-M345 into tubulin.

Colchicine was able to fit the colchicine binding site, showing a conformation identical to that of the X-ray structure of the colchicine (RMSD of 0.043 Å) (not shown), indicating that the docking protocol is reliable, and it could be used for the docking of the test compound. The RMSD value is inferior to 2 Å, usually considered a good threshold value for validating a structure for use in molecular docking. This is solid evidence that AutodockVina can predict docking poses accurately. Therefore, to further understand the possible binding mode of BP-M345 to tubulin, a careful inspection of the most stable docking pose of this small molecule was performed and compared to those obtained with controls. All tested compounds showed a binding mode very similar to the one of crystallized colchicine (Figure 9A,B). Indeed, docking studies revealed that BP-M345 and controls occupy the colchicine binding site of α,β-tubulin mostly buried in the β-unit (Figure 9A,B), with one of its 3,4,5-trimethoxyphenyl groups occupying the same position as the trimethoxyphenyl group (A-ring) of colchicine, as also observed for controls combretastatin A4 and podophyllotoxin. However, the binging pose of BP-M345, a symmetric compound, shows that the other trimethoxyphenyl group occupies the α subunit of tubulin (Figure 9A–C), providing extra anchoring points and strengthening the binding, which may potentiate its inhibitory effect on tubulin.

Most of the interactions between the controls colchicine, combretastatin A4, and podophyllotoxin with tubulin are mainly hydrophobic, being the 3,4,5-trimethoxyphenyl group buried in a hydrophobic pocket, with interactions established with residues αGln11, αAla12, αAsn101, and αGlu183 (Figure 9D). Moreover, for colchicine, the carbonyl group of the cycloheptatrienone ring is predicted to form a hydrogen interaction with αVal181 (Figure 9B). This residue was previously reported as important for the interaction of colchicine with α-tubulin [44]. For combretastatin A4, the *p*-hydroxyphenyl group is predicted to establish a hydrogen interaction with βVal238, which has been described as one of the important residues in the hydrophobic pocket in the tubulin-combrestatin A4 complex [45] (Figure 9B). In contrast, a hydrogen interaction is established between the methoxy group of the E-ring of podophyllotoxin and βCys241 (reported as important for interaction with tubulin [46], Figure 9B). For BP-M345, the trimethoxyphenyl group that occupied the same position as the trimethoxyphenyl group (A-ring) of colchicine also established hydrophobic interactions (Figure 9D) (such as βCys241, βLeu248, βAla250, βLeu255, βAla316, and βLys352).

The 3,4,5-trimethoxyphenyl moiety has been widely described as a relevant scaffold to design novel tubulin polymerization inhibitors [44,47,48]. Our docking studies support the potent ability shown by BP-M345 to disrupt the microtubule assembly by inhibiting tubulin polymerization, emphasizing the importance of the 3,4,5-trimethoxyphenyl moiety for the binding to tubulin with consequent disruption of the microtubule assembly by inhibiting tubulin polymerization.

## 3. Discussion

Antimitotic drugs, such as taxanes and vinca alkaloids, have been widely used in cancer therapy in recent years. However, there are some issues that need to be overcome, namely their side effects and the resistance mechanisms developed by cancer cells, motivating the need to discover new antimitotics [49]. Diarylpentanoids have been reported to exhibit antitumor activity by interfering with multiple biologic processes, including cell cycle [33,50]. Here, we uncover the cytotoxic mechanism of the diarylpentanoid BP-M345. We demonstrate that BP-M345 interferes with the stability of KT-MT attachments, which leads to chromosome congression defects in NCI-H460 tumor cells. Although most BP-M345-treated cells exhibited bipolar spindles, these spindles were less robust than those in untreated cells. BP-M345-treated cells chronically activate the SAC, resulting in mitotic arrest. A significant fraction of cells died in mitosis by apoptosis after a prolonged arrest. These results are consistent with the observation that, during a prolonged mitotic arrest, the cells accumulate apoptotic signals, leading to cell death [12,51]. However, other cells were able to exit mitosis. As BP-M345 was inducing misaligned chromosomes, it is possible that these cells exited mitosis with missegregated chromosomes, thereby generating aneuploid cells. Different cell fates are possible after missegregation errors. The cells might die in the following interphase if these errors are extensive, as we observed in the time-lapse assay in which a percentage of cells suffered post-mitotic death. Other cells might even become senescent or continue to proliferate [52]. In the time-lapse assay, we observed that a percentage of cells exited mitosis and survived during the period recorded (48 h). However, in the colony formation assay, when we treated the cells with BP-M345, no colonies were observed. This suggests that the cells that survived during the 48 h exposure to BP-M345 were unable to proliferate, possibly due to massive chromosome missegregation errors.

The mechanistic of the antimitotic activity of BP-M345 resembles that reported for other antimitotic agents such as paclitaxel, one of the most effective and widely used natural anticancer drugs [53]. Indeed, when cells are exposed to paclitaxel, they arrest in mitosis due to chronic activation of the SAC. The mitosis-arrested cells then undergo different fates: death in mitosis; unequal division into aneuploidy daughter cells; exit from mitosis without undergoing division (slippage), after which cells might then die in interphase, arrest in interphase, or enter additional cell cycles [53]. We recently confirmed this paclitaxel mechanistic in the NCI-H460 tumor cells [54]. We found that paclitaxel arrested NCI-H460 cells in mitosis for 155 min, less than the 218.7 min imposed by BP-M345 in the present study. The cell fates of paclitaxel- and BP-M345-treated cells were similar, except that most BP-M345-treated cells died in mitosis, while paclitaxel-treated cells died predominantly after cell division [54]. Therefore, our compound exhibits an antimitotic activity with a mechanistic that is comparable to that of routinely used antimitotic agents such as paclitaxel.

Importantly, we found that NCI-H460 cancer cells were more affected by BP-M345 treatment, in a long-term assay, comparatively to HPAEpiC non-tumor cells, suggesting a relevant degree of selectivity. This result is in agreement with the GI_50_ value found for HPAEpiC cells, which was 2.89-fold higher than in NCI-H450 (selectivity index), which precluded any comparison between the two cell lines as to the mechanistic of the compound, at the concentration that killed the tumor cells. The discovery of compounds with selective cytotoxicity that are able to distinguish between normal and tumor cells is a major challenge in cancer therapy, highlighting the potential of BP-M345 as a hit compound. In sum, here, we demonstrate that the diarylpentanoid BP-M345 exhibits a potent antitumor activity in vitro, acting as an antimitotic agent that induces massive cancer cell death, highlighting its potential as an antitumor agent that deserves to be further explored.

As a result of the presence of an α,β-unsaturated carbonyl system, BP-M345 might also behave as a Michael acceptor, which could react with multiple nucleophilic biomolecules, acting as a covalent binder. Nevertheless, it has been demonstrated that some BP-M345 analogues can undergo a reversible thia-Michael reaction, being regenerated diarylpentanoids [55]. Additionally, several diarylpentanoids have shown promising antitumor activity without any remarkable toxicity in in vivo assays [56,57]. Therefore, the presence of a α,β-unsaturated carbonyl system should not be regarded as a general knockout criterion that excludes this hit from further development. Taking this into account, further studies are required to confirm the antitumor potential of BP-M345 in animal models of cancer in order to evaluate its toxicity and to determine its effectiveness as potential antitumor agent. Considering that diarylpentanoids are well known for their effect in several targets involved in carcinogenesis, it will also be important to perform more studies to determine its exact molecular targets.

## 4. Material and Methods

### 4.1. Chemicals

Details concerning the synthesis of diarylpentanoid BP-M345 (Figure 1) were previously reported [33]. Briefly, an aqueous solution of 40% sodium hydroxide was added to a solution of tetrahydro-4H-pyran-4-one (100 mg, 0.99 mmol) in methanol until pH 13–14. Then, a solution of 3,4,5-trimethoxybenzaldehyde (421.2 mg, 2.99 mmol) in methanol was slowly added to the reaction mixture. The reaction was left at room temperature for 2 days and was monitored by TLC. After, crushed ice was added to the reaction mixture and neutralized with 5 M HCl solution. After the addition of crushed ice, the solution was extracted with ethyl acetate (3 × 50 mL), and the organic layer was collected, washed with water, dried over with anhydrous sodium sulfate, and concentrated under reduced pressure. The obtained residue was purified by crystallization from ethyl acetate (yield: 60%, as yellow solid). The structure elucidation of compound was established by 1H and 13C NMR techniques, and data were in accordance with the data previously reported [33]. BP-M345 was dissolved in n sterile DMSO (Sigma-Aldrich Co. Ltd., Gillingham, UK) to a stock concentration of 60 mM. Several aliquots were prepared and stored at −20 °C to preserve compound activity. For experiments, BP-M345 was diluted in fresh culture medium at desired concentrations.

### 4.2. Cell Lines and Culture Conditions

A375-C5 (melanoma), MCF-7 (breast adenocarcinoma) and NCI-H460 (non-small cell lung cancer) cell lines were obtained from European Collection of Cell Culture, UK and were grown in RPMI-1640 medium (Roswell Park Memorial Institute, Biochrom, Cambridge, UK), with 5% inactivated FBS (fetal bovine serum, Biochrom). The non-tumor human pulmonary alveolar epithelial cells (HPAEpiC) were obtained from ScienCell Research Laboratories and grown in DMEM medium (Dulbecco′s Modified Eagle′s, Biochrom) supplemented with 10% FBS and 1% non-essential amino acids (Sigma-Aldrich Co., Saint Louis, MO, USA). All cell lines were maintained in a humidified incubator (Hera Cell, Heraeus, Hanau, Germany) at 37 °C and with 5% CO_2_.

### 4.3. Sulforodamine B (SRB) Assay

A total of 5.0 × 10^4^ NCI-H460, A375-C5, and MCF-7 cells, or 6.0 × 10^4^ HPAEpiC cells were seeded with complete culture medium in 96-well plates and incubated for 24 h at 37 °C and 5% of CO_2_. A 96-well plate was used to quantify the cell count at time zero, and the cells were fixed with 50% (*m*/*v*) trichloroacetic acid (Merck Millipore, Darmstadt, Germany), for 1 hour at 4 °C and then washed with distilled water and left to dry. At the same time, in another 96-well plate, cells were treated with two-fold dilutions of BP-M345, with a range of 0 to 1.17 µM, or with Doxorubicin, used as a positive control, ranging from 0 to 0.07 µM. After 48 h, cells were fixed with 50% (*m*/*v*) trichloroacetic acid (Merck Millipore, Darmstadt, Germany) for 1 hour at 4 °C and then washed with distilled water and left to dry. Then, cells were stained with Sulforhodamine B (Sigma-Aldrich Co. Ltd., Gillingham, UK) for 30 min at room temperature and washed 5 times with 1% (*v*/*v*) acetic acid (Merck Millipore, Darmstadt, Germany) and left to dry. SRB complexes were then solubilized with 10 mM Tris buffer (Sigma-Aldrich Co. Ltd., Gillingham, UK) for 30 min, and the absorbance was measured at 515 nm in a microplate reader (Biotek Synergy 2, BioTek Instruments, Inc., Winooski, VT, USA). A dose–response curve was obtained for each cell line treated with BP-M345 and Doxorubicin, and the concentration that caused cell growth inhibition of 50% (GI_50_) was determined.

### 4.4. Mitotic Index Determination

A total of 9.0 × 10^5^ NCI-H460 cells were seeded with complete culture medium in 6-well plates for 24 h. Cells were then treated for 16 h with 0.74 µM of BP-M345 (2-fold GI_50_) or 1 μM of Nocodazole (Sigma-Aldrich Co. Ltd., Gillingham, UK) used as a positive control. Dimethyl sulfoxide (DMSO), up to 0.25% concentration, was included as compound solvent control. Then, at least 3000 cells were accounted for the determination of the mitotic index (MI), from random phase-contrast microscope fields. MI was calculated using the following formula: MI (%) = (number of mitotic cells/total number of cells) × 100.

### 4.5. Flow Cytometry

For cell cycle analysis, after 16 h treatment, NCI-H460 cells were harvested, washed twice in phosphate-buffered saline (PBS), and fixed in ice-cold 70% ethanol at 4 °C for 30 min. Then, cells were treated with 5 μg/mL of Propidium Iodide and 100 μg/mL of RNase in PBS for 30 min and analyzed in the flow cytometer.

For apoptosis detection, after 24 h treatment, floating and adherent cells were collected and processed with the “Annexin V-FITC Apoptosis Detection Kit” (eBioscience, Vienna, Austria) according to manufacturer’s instructions. Fluorescence was assessed by BD Accuri™ C6 Plus Flow cytometer (BD Biosciences, Qume Drive, San Jose, CA, USA), and data were analyzed with BD Accuri TM C6 Plus software, version 1.0.27.1 (www.bdbiosciences.com). For cell cycle and apoptosis analysis, at least 20,000 events per sample were collected.

### 4.6. Indirect Immunofluorescence

NCI-H460 cells were grown on poly-L-lysine-coated coverslips for 24 h. Then, cells were treated with 0.74 µM of BP-M345 and, after 16 h, were fixed with methanol (Sigma-Aldrich) for 10 min at −20 °C and washed 3 times with PBS for 5 min. After washing in PBS, cells were blocked with 10% FBS in 0.05% Tween-20 in PBS (PBST) for 30 min at room temperature. Cells were then incubated with primary antibodies diluted in 5% FBS in PBST, for 1 h. The following primary antibodies were used: human anti-CREST (1:4000, gift from E. Bronze-da-Rocha, University of Porto, Portugal); mouse anti-α-tubulin (1:2500, Sigma-Aldrich Co. Ltd., Gillingham, UK); and mouse anti-BUBR1 (1:200, Milipore Chemicon). Cells were then washed 3 times with PBST for 5 min and incubated for 1 h with Alexa Fluor 488 and 568 conjugated secondary antibodies (Molecular Probes, Eugene, OR, USA) diluted at 1:1500. DNA was stained with 2 µg/mL 4′,6-diamidino-2-phenylindole (DAPI, Sigma-Aldrich Co. Ltd., Gillingham, UK) diluted in Vectashield mounting medium (Vector, H-1000, Burlingame, CA, USA).

### 4.7. MG-132 Proteasome Inhibitor Assay

Cells were treated with 0.74 µM of BP-M345 for 16 h, followed by addition of 10 µM of the proteasomal inhibitor MG-132 (Sigma-Aldrich Co. Ltd., Gillingham, UK) for 1 h. Then, the immunofluorescence assay was performed using an antibody mouse anti-α-tubulin (1:2500, Sigma-Aldrich Co. Ltd., Gillingham, UK), and DAPI. The number of metaphases with full alignment and metaphases with misaligned chromosomes was quantified in 10 random microscope fields.

### 4.8. Cold Treatment Assay

NCI-H460 cells were prepared as in the MG-132 assay and then subjected at 4 °C for 5 min followed by immunofluorescence using the antibodies human anti-CREST (1:4000, gift from E. Bronze-da-Rocha, University of Porto, Portugal) and mouse anti-α-tubulin (1:2500, Sigma-Aldrich Co. Ltd., Gillingham, UK). The numbers of attached and free kinetochores were counted from at least 6 random cells.

### 4.9. Colony Formation Assay

Cells were resuspended in culture medium, NCI-H460 cells were seeded at 5.0 × 10^2^ cells/well, and HPAEpiC were seeded at 2.0 × 10^3^ cells/well, according to the respective growth kinetics, onto six-well plates, allowed to attach for 24 h. Then, cells were treated with 0.185 and 0.093 µM of BP-M345. Untreated and DMSO-treated cells were included as controls. Forty-eight hours later, cells were washed with PBS and maintained for 6 days in complete culture medium without drugs. Following 10 min of methanol fixation, colonies were stained with 0.05% crystal violet for 20 min and were counted to determine the efficiency of clonal formation (percentage of grown colonies from 500/2000 seeded cells). Only colonies with most than 50 cells were accounted for.

### 4.10. Live-Cell Imaging

For live-cell imaging experiments, 1.9 × 10^5^ NCI-H460 cells were seeded onto LabTek II chambered cover glass (Nunc, Penfield, NY, USA) containing RPMI, for 24 h at 37 °C with 5% CO_2_. Cells were then treated with 0.74 μM of BP-M345, and images were captured at 10 min intervals up to 48 h under differential interference contrast (DIC) optics, with a 63× objective on an Axio Observer Z.1 SD inverted microscope, equipped with an incubation chamber with the temperature set to 37 °C and an atmosphere of 5% CO_2_. Movies were generated from the time-lapse images using ImageJ software (version 1.51, Rasband, W.S., ImageJ, U.S. National Institutes of Health, Bethesda, MD, USA). The number of cells arrested at mitosis or in cell death was scored based on cellular morphology. Dead cells were classified into death in mitosis (DiM) or post-mitotic death (PMD) when death occurred during or following cell division, respectively.

### 4.11. Image Acquisition and Processing

For phase contrast microscopy, a Zeiss Primo Vert microscope (Carl Zeiss, Oberkochen, Germany) and a Nikon TE 2000-U microscope (Nikon, Amsterdam, The Netherlands) with a 10× objective were used. The Nikon microscope used a DXM1200F digital camera with Nikon ACT-1 software (Melville, NY, USA). For experiments where image acquisition was performed using fluorescence, an Axio Observer Z.1 SD microscope (Carl Zeiss, Germany) was used, coupled to an AxioCam MR3, and with the Plan Apochromatic 63×/NA 1.4 objective. The deconvolution was performed with the software AxioVision Release 4.8.2 SPC, and the images were processed using ImageJ version 1.51.

### 4.12. Statistical Analysis

Statistical analysis was performed using an unpaired Student’s *t*-test or two-way ANOVA with Tukey’s multiple comparisons test in the GraphPad Prism version 6 (GraphPad software Inc., San Diego, CA, USA). Data are presented as the mean ± standard deviation (SD) of three independent experiments and the level of statistical significance was established considering the probabilities of * *p* < 0.05, ** *p* < 0.01, *** *p* < 0.001 and **** *p* < 0.0001.

### 4.13. Virtual Screening and Docking Studies

The structures of diarylpentanoid BP-M345 and controls were drawn using ChemDraw 17.0. The three-dimensional (3D) structures of the diarylpentanoid and controls were minimized using the Austin Model 1 parameterization of the MNDO method (AM1) implemented in ArgusLab 4.0.1. The calculation was finished when the gradient between any two successive steps in the geometry search was less than 0.1 kcalA^−1^mol^−1^; the maximum number of geometry steps were set to 1000. The calculation run until the maximum steps were reached or the convergence criteria was met, whichever came first. The 3D structure of tubulin was obtained from Protein Data Bank (PDB id: 4O2B) and prepared for docking using AutoDockTools 1.5.7. Docking simulations between the tubulin and small molecules were undertaken in PyRx 0.8 using AutoDock Vina [58]. Docking was run using an exhaustiveness of 8, engulfing the cavity occupied by the crystallographic colchicine (PDB id: 4O2B) [59]. Nine conformations for each ligand were obtained. The top ranked conformations of BP-M345 and controls were further analyzed concerning noncovalent interactions using Pymol 2.2.4 [60] and MOE v2014 [61].

## 5. Conclusions

In this work, it was confirmed that BP-M345 displayed potent tumor antiproliferative activity with high selectivity index and valuable data on the characterization of the mechanism of action was presented. This diarylpentanoid promotes a prolonged SAC-dependent mitotic arrest by interfering with mitotic spindle assembly, followed by massive apoptosis. The overall results indicate that the diarylpentanoid BP-M345 exerts its antiproliferative activity by inhibiting mitosis through microtubule perturbation and causing cancer cell death, highlighting its potential as antitumor agent.

## Figures and Tables

**Table 1:** Tumor cell growth inhibitory activity of compound BP-M345.

		GI_50_ (μM) ^1^	
	A375-C5	MCF-7	NCI-H460
BP-M345	0.24 ± 0.01	0.45 ± 0.06	0.37 ± 0.00
Doxorubicin	0.030 ± 0.04	0.028 ± 0.01	0.028 ± 0.01

^1^ GI_50_ represents the concentration that causes 50% cell growth inhibition at 48 h. Doxorubicin was used as positive control. The results are expressed as mean ± standard deviation from three independent experiments.

**Table 2:** Selectivity index of BP-M345.

	HPAEpiC ^1^	Selectivity Index ^2^		
	GI_50_ (μM)	A375-C5	MCF-7	NCI-H460
BP-M345	1.07 ± 0.16	4.45	2.38	2.89
Doxorubicin	0.054 ± 0.012	1.81	1.91	1.95

^1^ The GI_50_ from the non-cancer cells HPAEpiC was calculated as described above; ^2^ Selectivity Index: GI_50_ on non-cancer cells/GI_50_ on cancer cells. Doxorubicin was used as positive control for selectivity index.

**Table 3:** Docking scores (Kcal mol^−1^) on tubulin target for the diarylpentanoid BP-M345 and controls colchicine, combretastatin A4, and podophyllotoxin.

Ligand	Docking Score (kcal mol^−1^)
Colchicine	−10.1
Combretastatin A4	−7.8
Podophyllotoxin	−8.4
BP-M345	−8.7

## Supplementary Materials

The following are available online, Video S1: Untreated cell from time-lapse imaging (DIC microscopy), available online at https://youtu.be/vWWhmPv5_k0, demonstrating normal mitosis. Video S2: BP-M345-treated cell from time-lapse imaging (DIC microscopy), available online at https://youtu.be/XW0bodY5zYk, showing a mitotic arrest followed by death in mitosis. Video S3: BP-M345-treated cell from time-lapse imaging (DIC microscopy), available online at https://youtu.be/aPWBxY5D87M, showing a cell exiting mitosis followed by cell death in interphase. Figure S1: Dose–response curves of BP-M345 treatment from three independent experiments of NCI-H460, A365-C5, MCF-7 and HPAEpiC cell lines. The error bars represent mean ± SD. GI_50_ represents the concentration that causes 50% cell growth inhibition at 48 h.

## Sample Availability

Samples of the compound BP-M345 are available from the authors.
