# Supplementary material for: BP-M345, a New Diarylpentanoid with Promising Antimitotic Activity
Source: Molecules. 2021 Nov 25;26(23):7139. doi: 10.3390/molecules26237139 (PMC8659070; doi:10.3390/molecules26237139)
Supplement: Supplementary file 1 [file molecules-26-07139-s001.zip › molecules-1444891-supplementary.pdf]

Supplementary Materials

BP-M345, a new diarylpentanoid with promising antimitotic activity

Pedro Novais[1,2,3,#], Patrícia M. A. Silva[1,5#], Joana Moreira[6,7], Andreia Palmeira[6,7], Isabel Amorim[4], Madalena Pinto[6,7], Honorina Cidade[6,7,*] and Hassan Bousbaa[1,*]

1 CESPU, Institute of Research and Advanced Training in Health Sciences and Technologies (IINFACTS), Rua Central de Gandra, 1317, 4585-116 Gandra, Portugal; pedro.ha.novais@gmail.com (P.N.); patricia.silva@cespu.pt (P.M.A.S).
2 Faculty of Sciences, University of Porto, Rua do Campo Alegre, s/n, 4169-007 Porto, Portugal
3 ICBAS, Instituto de Ciências Biomédicas Abel Salazar, University of Porto, Porto, Portugal.
4 GreenUPorto (Sustainable Agrifood Production) Research Center, Faculty of Sciences, University of Porto, Rua do Campo Alegre, s/n, 4169-007 Porto, Portugal; mpamorim@fc.up.pt (I.A.).
5 TOXRUN – Toxicology Research Unit, University Institute of Health Sciences, CESPU, CRL, 4585-116 Gandra, Portugal.
6 Laboratory of Organic and Pharmaceutical Chemistry, Department of Chemical Sciences, Faculty of Pharmacy, University of Porto, Rua de Jorge Viterbo Ferreira 228, 4050-313 Porto, Portugal; up201302558@edu.ff.up.pt (J.M.); apalmeira@ff.up.pt (A.P.); madalena@ff.up.pt (M.P.).
7 Interdisciplinary Centre of Marine and Environmental Research (CIIMAR), University of Porto, Edifício do Terminal de Cruzeiros do Porto de Leixões, Avenida General Norton de Matos, S/N, 4450-208 Matosinhos, Portugal.
# Authors contributed equally to this work
* Correspondence: hcidade@ff.up.pt (H.C.); Tel.: +351-220428688 (H.C.); hassan.bousbaa@iucs.cespu.pt (H.B.) Tel.: +351-220428688 (H.C.); +351- 224157186 (H.B.).

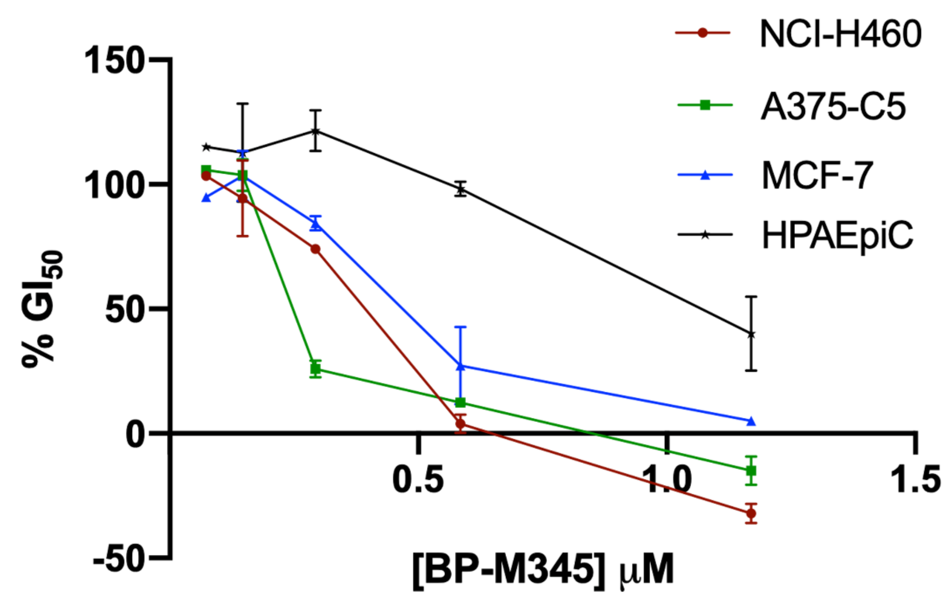

Figure S1. Dose-response curves of BP-M345 treatment from three independent experiments of NCI-H460, A365-C5, MCF-7 and HPAEpiC cell lines. The error bars represent mean ± SD. GI_{50} represents the concentration that causes 50% cell growth inhibition at 48 hours.
